# Egg deposition of maternal testosterone is primarily controlled by the preovulatory peak of luteinizing hormone in Japanese quail

**DOI:** 10.1016/j.ygcen.2017.05.004

**Published:** 2018-01-15

**Authors:** Monika Okuliarova, Simone L. Meddle, Michal Zeman

**Affiliations:** a Department of Animal Physiology and Ethology, Faculty of Natural Sciences, Comenius University, Bratislava, Slovak Republic; b The Roslin Institute, The Royal (Dick) School of Veterinary Studies, The University of Edinburgh, Easter Bush, UK; c Institute of Animal Biochemistry and Genetics, Slovak Academy of Sciences, Ivanka pri Dunaji, Slovak Republic

**Keywords:** Maternal effects, Yolk androgens, Ovulatory cycle, Luteinizing hormone, Quail

## Abstract

Differential transfer of maternal testosterone (T) into egg yolk provides a means of adjusting an offspring’s phenotype to ambient environmental conditions. While the environmental and genetic driven variability in yolk T levels is widely described, the underlying mechanisms are poorly understood. Here, we investigated whether neuroendocrine mechanisms controlling ovulatory processes are associated with the regulation of yolk T deposition. Circulatory profiles of luteinizing hormone (LH), T and estradiol levels were analysed during the last 7 h before ovulation in Japanese quail selected for contrasting yolk T concentrations. Moreover, the pituitary responsiveness to a single challenge with gonadotropin releasing hormone (GnRH) was evaluated. High egg T (HET) birds displayed higher concentrations of LH at 3.5 h before ovulation than low egg T (LET) birds while no differences were found around the time of expected ovulation. The pre-ovulatory profile of T and estradiol levels did not differ between LET and HET females but pre-ovulatory plasma T positively correlated with LH concentrations at 6.5 h and 3.5 h before ovulation. The LH response to GnRH did not differ between LET and HET females. Our results demonstrate that the pre-ovulatory LH surge can determine the amount of T transferred into the egg yolk. This link between yolk T deposition and the ovulatory cycle driven variation of reproductive hormones may explain balance between the effects of circulating T on female’s reproductive physiology and yolk T on offspring phenotype.

## Highlights

• Pre-ovulatory LH surge can control variability in yolk T levels.
• High yolk T deposition was not reflected in permanently increased plasma T levels.
• Plasma LH response to GnRH did not explain differences in pre-ovulatory LH surge.

## 1 Introduction

Birds lay eggs with significant amounts of yolk androgens, which are transferred during the rapid growth of ovarian follicles (Okuliarova et al., 2010). Due to the maternal origin of yolk hormones they have been considered to serve as mediators of trans-generational maternal effects. From an adaptive point of view this means that differential deposition of yolk androgens may programme phenotypic development of offspring promoting faster adaptations to environmental conditions experienced by the mother (Groothuis et al., 2005, von Engelhardt and Groothuis, 2011). Indeed, embryonic exposure to experimentally elevated testosterone (T) in the egg prior to incubation has been shown to influence a number of physiological and behavioural offspring traits, including growth (Cucco et al., 2008, Schwabl, 1996), immune functions (Sandell et al., 2009, Tobler et al., 2010) and competitive abilities (Muller et al., 2012a; Muller et al., 2009).

High inter-female variability in yolk T deposition has been found within different avian species. Inter-female differences are associated with environmental and social factors encountered by the female during the breeding season, such as mating partner (Garcia-Fernandez et al., 2010), breeding density (Pilz and Smith, 2004), immunological load (Gil et al., 2006, Tschirren et al., 2004), food availability (Benowitz-Fredericks et al., 2013) and stressful events (Henriksen et al., 2011, Okuliarova et al., 2010) and with female’s age as well (Guibert et al., 2012). However, under stable environmental and social conditions females clearly differ in their yolk T concentrations showing high genetically determined variability in this trait. Heritable variation of yolk T levels was recently evidenced by a selection experiment in precocial Japanese quail (Okuliarova et al., 2011) as well as by a mother-daughter regression approach in altricial great tits and canaries (Muller et al., 2012b; Tschirren et al., 2009). Despite the fact that numerous factors influencing yolk T deposition have already been identified, the physiological mechanisms behind this environmentally and genetically driven variability in yolk T levels are still to be elucidated.

In avian females, biosynthesis of sex steroids including T is located in cell layers comprising the wall of ovarian follicles (Robinson and Etches, 1986). From inner to outer side, progesterone, androgens and estradiol are produced in cells of granulosa, theca interna and theca externa, respectively (Porter et al., 1989). Steroidogenic capacity of yellow hierarchical follicles as well as their sensitivity to gonadotropins changes during the follicular maturation. Estradiol is mainly produced in the small follicles, T in the third and second follicles and progesterone in the largest pre-ovulatory follicles (Bahr et al., 1983, Robinson and Etches, 1986).

From follicular cells, sex steroids pass both into the maternal circulation and into the oocyte. During breeding, sex hormones control female reproductive physiology and behaviour through the hypothalamic-pituitary-gonadal (HPG) axis (Deviche, 2015, Johnson, 2015). Therefore, common neuroendocrine mechanisms are expected to regulate both female’s reproduction and yolk T deposition. This issue has been recently studied based on the gonadotropin-releasing hormone (GnRH) challenge to pre-laying female songbirds showing a positive correlation between the magnitude of response in maternal plasma T levels and yolk T concentrations in laid eggs (Jawor et al., 2007, Muller et al., 2011). Similar findings were obtained following repeated GnRH injections to female Japanese quail, which continuously laid one egg per day (Peluc et al., 2012). Moreover, in house sparrows, GnRH stimulation simultaneously increased both circulating T levels and yolk T in pre-ovulatory ovarian follicles, although the correlation between an increase in plasma T and yolk T of laid eggs was not recorded (Egbert et al., 2013). Together, these studies indicate that factors stimulating the release of GnRH may influence female reproductive performance as well as yolk T mediated maternal effects on offspring’s phenotype. However, responsiveness to GnRH is not sufficient to explain direct physiological mechanisms responsible for the amount of yolk T in the egg. Moreover, considering baseline plasma T concentrations, the authors of the aforementioned studies did not find any correlation between the initial plasma and yolk T levels. Interestingly, in quail selected for contrasting yolk T concentrations, this trait was not correlated with plasma T levels implying that both yolk and plasma T are independently controlled (Okuliarova et al., 2011).

In laying females, the production of sex hormones is tightly related to the ovulatory cycle, which occurs at a daily basis in domestic fowl. In this way, T markedly varies in the circulation showing a distinct pre-ovulatory surge, which precedes the pre-ovulatory peaks of both progesterone and luteinizing hormone (LH) (Etches and Cheng, 1981, Johnson and van Tienhoven, 1980). The pre-ovulatory peak of estradiol coincides with the T surge and is needed to prime the pituitary for progesterone positive feedback on LH release (Johnson and van Tienhoven, 1980, Wilson and Sharp, 1976). Moreover, since T is converted to estradiol by aromatisation, changes of plasma estradiol levels may accompany differential yolk T deposition. Therefore, we hypothesized that the pre-ovulatory increase of reproductive hormones would represent a direct determinant of yolk T deposition. To evaluate this prediction we compared circulatory profiles of LH, T and estradiol levels during last 6–7 h before ovulation in female Japanese quail, which were selected for contrasting yolk T concentrations and thus showed consistent differences in amounts of this androgen in their eggs. Moreover, we analysed whether yolk T deposition is related to a pituitary responsiveness to a single GnRH challenge in these females.

## 2 Material and methods

### 2.1 Animals

The study was performed in Japanese quail (*Coturnix japonica*) that were selected for contrasting yolk T concentrations for six generations (Okuliarova et al., 2011). Quail were bred and kept at the Institute of Animal Biochemistry and Genetics, the Slovak Academy of Sciences (IABG), Ivanka pri Dunaji, the Slovak Republic under a light/dark cycle of 14L:10D (lights on at 6 am). Food (mash for laying hens) and water were provided *ad libitum*. Yolk T concentrations were measured in all females 2 months before they were included into the study. Three eggs per female were analysed and mean (±standard error) yolk T levels were 8.4 ± 0.5 pg/mg and 16.1 ± 1.1 pg/mg for low (LET line) and high (HET line) egg T females, respectively. It is not likely that such line differences changed over a 2-month period since there is high within-female repeatability in yolk T levels across different stages of the first reproductive cycle in Japanese quail (Okuliarova et al., 2009). All experiments were conducted in quail showing a regular ovulatory cycle at the age of 6–7 months. Specific housing conditions in each experiment are described below.

Animal care and the experiments were conducted in an approved breeding facility (SK PC 7010 Np) and in accordance with laws and regulations of the Slovak Republic and approved by the Ethical Committee of the IABG.

### 2.2 Experiment 1

Thirty-one breeding pairs of one male and one female (15 LET; 16 HET) were included in Experiment 1 and housed in single cages. To estimate timing of an ovulatory cycle, oviposition was daily recorded for each female over one week-period. All cages were continuously monitored by videocamera and time of oviposition was exactly recorded once the egg appeared on the screen. Consequently, the time of ovulation was calculated by adding 0.5 h to the time of oviposition since the ovulation is expected to occur within 30 min after egg laying in Japanese quail (Woodard and Mather, 1964). Therefore, all data in our study are reported as hours before anticipated ovulation.

Serial blood samples were collected from each female three times before ovulation. Exact ranges of sampling times were 7.8–5.4, 4.8–2.4, and 0.6–0.2 h before anticipated ovulation and we use rounded mean times 6.5, 3.5 and 0.5 h throughout the text, respectively. Blood was always taken from the wing vein using heparinised syringes. Samples of females that did not lay egg at the expected time were not included in analyses. Plasma was separated by centrifugation and stored at −20 °C. Within the interval of 3 days before blood sampling, 2–3 eggs (40 LET; 40 HET) were collected per each female and yolk T levels were determined.

### 2.3 Experiment 2

Fifteen females (8 LET; 7 HET) were used in Experiment 2, in which the pituitary responsiveness to GnRH challenge was analysed. Quail were housed in groups containing 4–5 females per cage and they were moved to individual cages during the experiment. The GnRH challenge was performed with an intramuscular injection of a synthetic analogue of GnRH (Supergestran NORDIC Pharma, Czech Republic) at a dose of 1.5 µg per 100 g body weight in 100 µl of saline. Although the individual time of oviposition was not recorded, based on the mean egg laying time we estimated that the quail were challenged with GnRH between 5 and 6 h before the expected ovulation. Moreover, we checked that all females had calcified egg in the uterus at the time of sampling to ensure that they were in the same stage of their pituitary responsiveness to GnRH (Burke and Cogger, 1977, Connolly and Callard, 1988). Blood samples were taken immediately prior to the GnRH injection and then 5 and 15 min post-injection in the same way as described in Experiment 1. To ensure quick manipulation and exact time of sampling, quail were placed in a dark box between the first and second sampling and they were moved back into their cages between the second and third sampling. Plasma was separated by centrifugation and stored at −20 °C.

### 2.4 Hormone assays

Yolk T concentrations were analysed after ether extraction by radioimmunoassay (RIA). Extraction procedure followed the previously published protocol (Okuliarova et al., 2011). Briefly, yolk subsamples (100 mg of yolk diluted 10 times with deionised water) were extracted twice with 2 mL of a mixture of diethyl and petroleum ether (7:3). The samples were centrifuged, snap-frozen and ether phases were decanted and dried under nitrogen. The extracts were precipitated in 2 mL of 70% methanol for 2 days at −20 °C. Thereafter, the samples were centrifuged, decanted, and dried under nitrogen. Dried extracts were reconstituted in 0.3 mL of phosphate-buffered saline. The mean (±SE) extraction recovery was 78.0 ± 0.6%.

Plasma LH, T and estradiol levels were measured by direct radioimmunoassays (RIAs). To determine LH, a micromodification of RIA was used as described previously (Sharp et al., 1987). This RIA has been used to quantify plasma LH in various avian species including quail (Davies et al., 2015, Maung and Follett, 1978, Meddle et al., 2006). All samples were measured in 20-uL duplicates in a single assay with the intra-assay variation coefficient of 24.8% and the minimum detectable limit of 0.2 ng/mL.

Testosterone concentrations were measured in both plasma (20 μL aliquot) and yolk extracts (10 μL aliquot) using [1,2,6,7-3H]-testosterone (PerkinElmer, USA, specific activity 63.47 Ci/mmol) and a specific antibody generated in rabbits against testosterone-3-(carboxy-methyl)oxime-bovine serum albumin conjugate. Bound and free T fractions were separated by dextran-coated charcoal and centrifuged at 3000*g* at 4 °C for 10 min. Yolk and plasma samples were measured separately in three assays with intra-assay variation coefficients of 7.2%, 13.3% and 3.6%, respectively. Inter-assay variation coefficient for plasma T was 9.8%. Assay sensitivity was 1.3 pg per tube.

Plasma estradiol concentrations were measured by commercial ^125^I labelled RIA kit for 17β-Estradiol (RIA-0220, DRG Instruments GmbH, Germany) according to the manufacturer’s instructions. All samples were measured in a single assay with intra-assay variation coefficient of 7.2%. Assay sensitivity was 3.88 pg/mL.

### 2.5 Statistical analyses

All data were examined for a normal distribution by the Kolmogorov-Smirnov test. Yolk T concentrations and plasma LH levels in Experiment 2 showed a deviation from normality and therefore they were logarithmically transformed. Hormonal changes during the ovulatory cycle and in response to GnRH challenge were analysed with the repeated measures analysis of variance (ANOVA) followed by Fisher’s Least Significant Difference *post hoc* tests. Yolk hormone levels were analysed using mixed general linear model with fixed factor of line and random factor of female identity nested within the line. Within-female variability in yolk T levels among eggs laid during 3 days before examination of pre-ovulatory hormone levels was evaluated by repeatability calculation (Lessells and Boag, 1987). Laying rate and time of expected ovulation were compared between LET and HET females by independent *t*-tests.

## 3 Results

### 3.1 Yolk T levels

As expected, eggs from HET females contained twice as high mean (± SE) yolk T concentrations than eggs from LET females (20.9 ± 1.3 *vs.* 10.3 ± 0.5 pg/mg; *F*_(1,49)_ = 43.66, *p* < 0.001). The same was found for the yolk T content (57.3 ± 3.3 *vs.* 26.9 ± 1.4 ng/yolk for HET and LET eggs, respectively; *F*_(1,49)_ = 32.13, *p* < 0.001). High repeatability of yolk T concentrations was calculated among eggs, which were collected per female within the interval of 3 days before examination of pre-ovulatory plasma hormone levels (*R* = 0.82; *F*_(30,49)_ = 12.51, *p* < 0.001).

### 3.2 Hormonal profiles during the ovulatory cycle

**Fig. 1:**
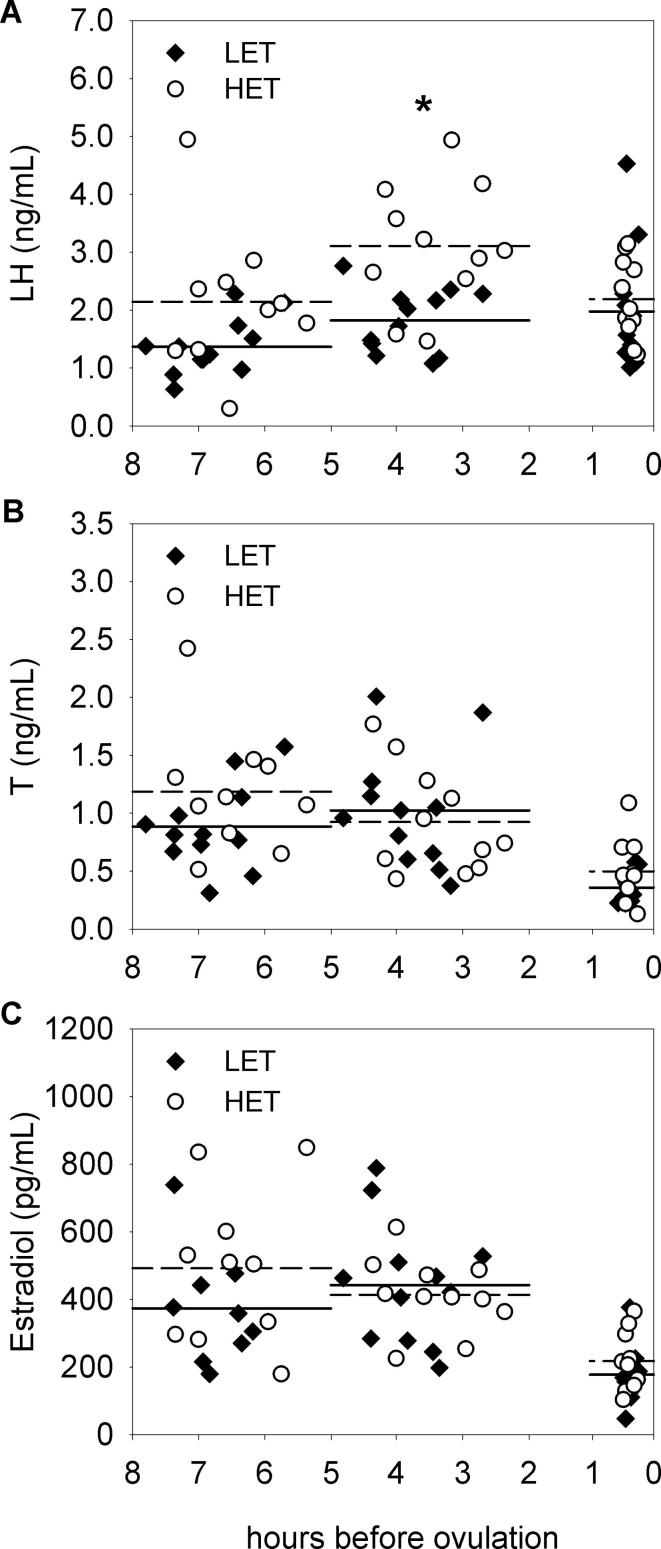
Plasma concentrations of A) luteinizing hormone – LH, B) testosterone – T and C) estradiol during the ovulatory cycle in low (LET, n = 12) and high (HET, n = 11) egg T female Japanese quail. Mean hormone levels for the sampling time 6.5, 3.5 and 0.5 h before ovulation are represented by solid and dashed lines in the LET and HET females, respectively. Sample size is 67, 68 and 63 for LH, T and estradiol levels. Asterisk denotes significant differences between LET and HET females at the level *p* < 0.05.

**Fig. 2:**
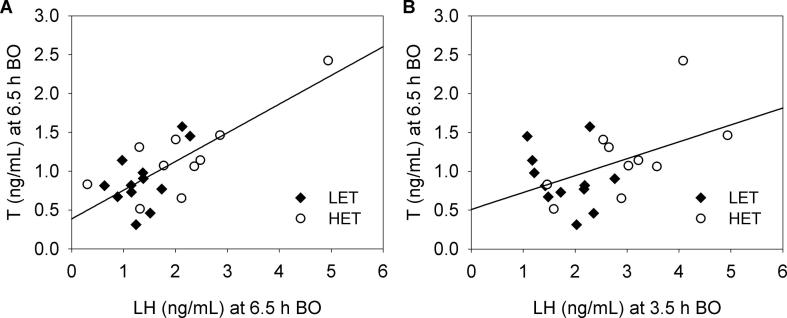
Relationship between plasma testosterone (T) at 6.5 h before ovulation (BO) and luteinizing hormone (LH) levels at A) 6.5 h and B) 3.5 h before ovulation in low (LET) and high (HET) egg T female Japanese quail.

Profile of plasma LH levels significantly differed between LET and HET females during the last 6–7 h before ovulation (line × preovulatory time interaction: *F*_(2,42)_ = 4.52, *p* < 0.05; Fig.1A). In HET females, the highest LH levels were found at 3.5 h before ovulation and this peak was significantly different from the levels at 6.5 (*p* < 0.01) and 0.5 h (*p* < 0.001) before ovulation. In LET females, LH concentrations increased from 6.5 to 0.5 h before ovulation (*p* < 0.05). Moreover, HET females displayed significantly higher LH levels than LET females at 3.5 h before ovulation (*p* < 0.05) with no line differences around the time of expected ovulation (Fig.1A). Both, plasma T and estradiol concentrations significantly varied in the same way during the last 6–7 h before ovulation (for T: *F*_(2,42)_ = 20.50, *p* < 0.001; Fig.1B and for estradiol: *F*_(2,40)_ = 32.29, *p* < 0.001; Fig.1C). They showed a decline from 3.5 to 0.5 h before ovulation (*p* < 0.001 for T and estradiol). No line differences (for T: *F*_(1,21)_ = 1.04, *p* = 0.319 and for estradiol: *F*_(1,20)_ = 0.77, *p* = 0.390) and the effects of interaction (for T: *F*_(2,42)_ = 1.86, *p* = 0.169 and for estradiol: *F*_(2,40)_ = 2.38, *p* = 0.105) were found. Plasma T levels at 6.5 h before ovulation showed significant positive correlation with LH concentrations at both 6.5 h (Pearson r = 0.76, N = 22, *p* < 0.001; Fig.2A) and 3.5 h (Pearson r = 0.47, N = 22, *p* < 0.05; Fig.2B) before ovulation.

Females with low and high yolk T eggs did not differ in their laying rate (LET: 91.4% and HET: 87.5%; *t*_29_ = 0.989, *p* = 0.331) and time of expected ovulation (LET: 5:22 pm and HET: 4:50 pm; *t*_21_ = 0.940, *p* = 0.358).

### 3.3 GnRH challenge

**Fig. 3:**
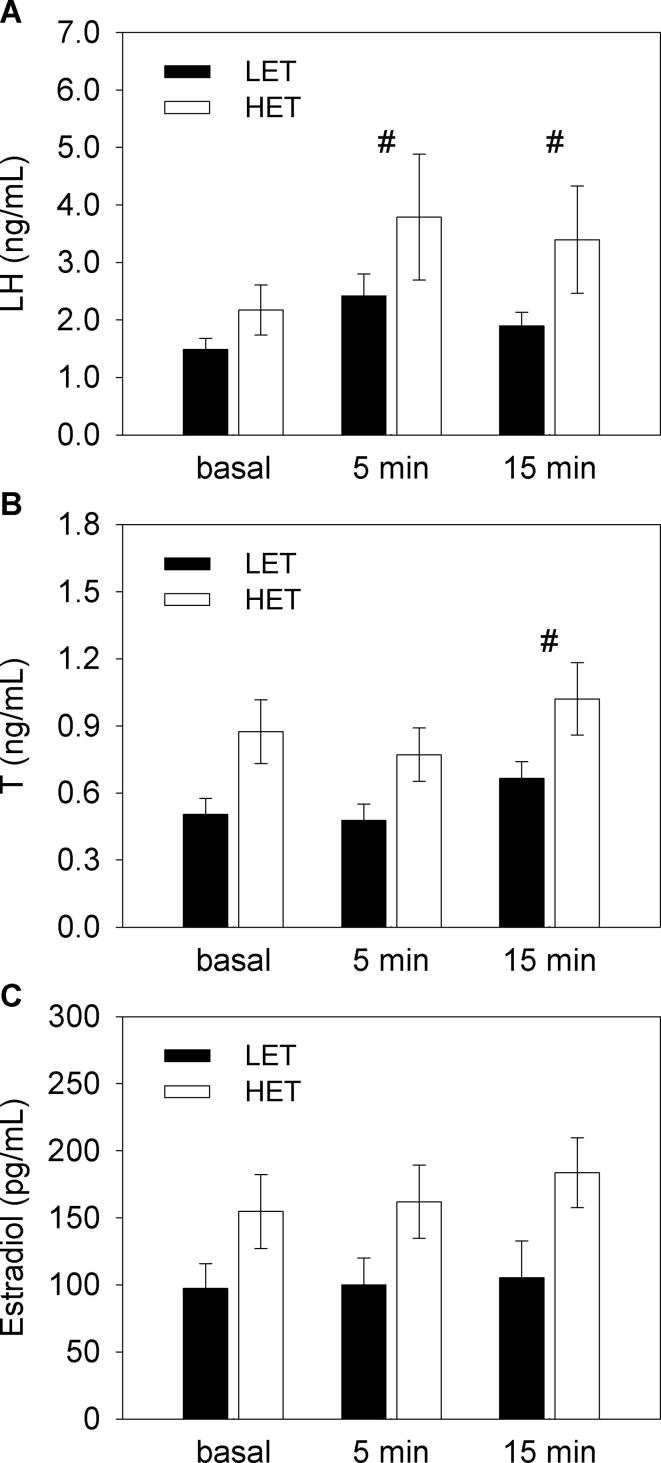
Plasma concentrations of A) luteinizing hormone – LH, B) testosterone – T and C) estradiol following a single GnRH injection to low (LET, n = 8) and high (HET, n = 7) egg T female Japanese quail. ^#^ denote significant increase of post GnRH hormone levels as compared to basal concentrations at the level *p* < 0.01. Significant differences between LET and HET females were detected for T (*p* < 0.05).

Plasma LH levels significantly increased over 15 min following GnRH stimulation (*F*_(2,26)_ = 15.45, *p* < 0.001; Fig.3A) and there was no effects of line (*F*_(1,13)_ = 2.39, *p* = 0.146) and an interaction between line and time (*F*_(2,26)_ = 0.51, *p* = 0.608). As compared to basal levels, LH concentrations were higher at both 5 min (*p* < 0.001) and 15 min (*p* < 0.01) post GnRH challenge. In response to GnRH stimulation, plasma T levels significantly increased at 15 min post injection (*F*_(2,26)_ = 11.33, *p* < 0.001; Fig.3B) while estradiol concentrations did not change (*F*_(2,26)_ = 1.42, *p* = 0.261; Fig.3C). Moreover, HET females displayed higher plasma T (*F*_(1,13)_ = 5.56, *p* < 0.05) and a tendency towards significantly higher estradiol levels (*F*_(1,13)_ = 4.18, *p* = 0.062) than LET females. No effects of interaction between line and time were detected for both T (*F*_(2,26)_ = 0.36, *p* = 0.702) and estradiol *(F*_(2,26)_ = 0.47, *p* = 0.627).

## 4 Discussion

In the present study, we examined whether pre-ovulatory changes of plasma LH, T and estradiol levels are associated with yolk T deposition in Japanese quail. Females laying eggs with high yolk T had significantly higher concentrations of LH at 3.5 h before ovulation than quail with low yolk T levels; while no differences were found around the time of expected ovulation. Pre-ovulatory plasma T levels at 6.5 h before ovulation positively correlated with LH concentrations at 6.5 h and 3.5 h before ovulation but high yolk T deposition in HET females was not reflected in consistently elevated T in their circulation.

In avian species, ovulation is regulated by a pre-ovulatory surge of reproductive hormones, mainly by the positive feedback between LH and progesterone (Etches and Cunningham, 1976, Johnson et al., 1985). Here our data showed for the first time that the pre-ovulatory increase of LH may determine the amount of T transferred into the egg yolk since quail with high yolk T levels displayed higher pre-ovulatory LH concentrations than females with low T content in their eggs. This finding suggests that the physiological mechanisms behind inter-individual differences in yolk T deposition involve a variation of plasma LH levels during the ovulatory cycle. Peripheral concentrations of LH typically reach a maximum by 3–6 h before ovulation in the domestic hen (Johnson and van Tienhoven, 1980) and quail (Doi et al., 1980, Gulati et al., 1981). However, in contrast to hens, which usually lay eggs in the morning, quail lay eggs in the afternoon (Houdelier et al., 2002). In our study, based on the time of oviposition, mean ovulation time occurred 3 h before lights off and did not differ between LET and HET quail. Corresponding to the pre-ovulatory profile of LH levels reported in quail (Gulati et al., 1981), HET females showed plasma LH peak at 3.5 h before ovulation followed by its decline at the time of ovulation. Surprisingly, this pattern was not found in LET females. Concentrations of LH remained high around the ovulation time implying that ovulation was either preceded by a narrow LH peak, which we most probably missed with our sampling times or LH did not decrease. Therefore, it is possible that beside the levels of pre-ovulatory LH peak also timing of LH surge may play a role in yolk T deposition.

Ovulatory cycle dependent variation of reproductive hormones is reflected also in plasma surges of T and estradiol, which usually precede pre-ovulatory peaks of LH and progesterone in birds (Doi et al., 1980, Etches and Cheng, 1981). In our study, plasma T and estradiol showed expected changes during the period of 6–7 h before ovulation. The pre-ovulatory increase of both hormones occurred between 3 and 6 h before ovulation and was followed by a decline at the expected ovulation time. This pattern is in line with data reported in quail and domestic fowl (Doi et al., 1980, Johnson and van Tienhoven, 1980) and did not differ between LET and HET females. Moreover, in contrast to pre-ovulatory LH levels, quail with high egg T did not display significantly higher pre-ovulatory concentrations of plasma T and estradiol as compared to LET females. Nevertheless, positive correlations between pre-ovulatory LH and T concentrations were found. In our study, we cannot exclude that due to individual differences the pre-ovulatory peaks of T and estradiol occurred outside our sampling interval in some individuals and thus we were not able to detect line differences. Indeed, it was reported that both hormones start to increase 8 h before ovulation (Doi et al., 1980). This possibility can be supported by the results obtained from Experiment 2, in which baseline plasma T levels were higher in HET than LET females and estradiol levels showed the same tendency.

The primary source of sex steroids is provided by cells in the wall of ovarian follicles, which steroidogenic capacity and sensitivity to gonadotropins change during the follicular maturation (Etches, 1996). Testosterone is produced mainly by middle-sized hierarchical follicles (Bahr et al., 1983) reflecting the maximum accumulation in the oocyte during the second follicular stage as was demonstrated in quail (Okuliarova et al., 2010). In more detail, our current data suggest that within 24 h cycle there is a distinct period before ovulation, during which the prevalent yolk T accumulation may occur. This is in agreement with T profile of the theca and granulosa layers showing significant variation during the ovulatory cycle with an increase at 6 h before ovulation (Bahr et al., 1983). Interesting implications arise from this ovulatory cycle driven variation of reproductive hormones, since it can help to understand how balance between the effects of circulating T on female’s reproductive physiology and yolk T on offspring phenotype is attained (Muller et al., 2007, Tobler and Smith, 2010).

It has been considered that the control of yolk T deposition can be either coupled with or independent of plasma T levels in the maternal circulation leading to either positive or no correlation between each other, respectively (Groothuis and Schwabl, 2008). As a consequence of the former, it is assumed that females, laying eggs with high T concentrations, by themselves will be exposed to high plasma T levels, which in turn can manifest by negative effects on female’s reproductive performance. Such effects were mainly demonstrated by an exogenous elevation of plasma androgen levels in some passerines (de Jong et al., 2016, O'Neal et al., 2008, Rutkowska et al., 2005). However, in line with our previous study (Okuliarova et al., 2011), HET quail with high yolk T deposition do not consistently display increased T levels in the circulation. Therefore, it is unlikely that there are any acute costs of high plasma T. In the context of the ovulatory cycle, a physiological role of pre-ovulatory T surge has been recently shown by its ability to indirectly influence pre-ovulatory peak of LH through a stimulation of granulosa cell progesterone production in the largest hierarchical follicle (Rangel et al., 2007, Rangel et al., 2009).

In the second experiment we asked whether differences in pre-ovulatory LH levels between LET and HET females could be explained by a different sensitivity of the pituitary gland to GnRH. Exogenous GnRH injection increased plasma LH levels 5 min after the stimulation but no differences in the pituitary responsiveness between LET and HET females were found. Therefore, this regulatory step of HPG axis does not seem to account for a different profile of pre-ovulatory LH levels as well as a genetically programmed variability of yolk T concentrations.

In several avian species, a female’s capacity to produce androgens measured as the magnitude of plasma T response to GnRH stimulation (Egbert et al., 2013) was positively associated with egg yolk T concentrations (Jawor et al., 2007, Muller et al., 2011, Peluc et al., 2012). In our study, although plasma T levels significantly increased 15 min post GnRH challenge, this probably did not reflect maximum T response. Our experiment was primarily designed to analyse the pituitary responsiveness and as a result the 15 min time interval post GnRH stimulation was not perfectly positioned to see peak ovarian steroid production (Peluc et al., 2012, Rosvall et al., 2013). There were no differences between LET and HET females in plasma T response to GnRH but overall circulatory T levels were higher in HET than LET quail. We expect that in line with Experiment 1 these differences could be attributed to the pre-ovulatory changes of reproductive hormones.

## 5 Conclusions

Our results provide significant insight into the physiological mechanisms, which underlie variability in yolk T deposition and consequently have an impact on maternal T dependent offspring’s phenotype. We have demonstrated that the levels of pre-ovulatory LH surge determine the amounts of T transferred into the egg yolk in Japanese quail. Moreover, we suggest that such link between yolk T deposition and ovulatory cycle driven variation of reproductive hormones enables birds to lay eggs with high amounts of yolk T without experiencing permanently elevated circulating T levels.
